# Prevention of lipopolysaccharide-induced preterm labor by the lack of CX3CL1-CX3CR1 interaction in mice

**DOI:** 10.1371/journal.pone.0207085

**Published:** 2018-11-06

**Authors:** Mika Mizoguchi, Yuko Ishida, Mizuho Nosaka, Akihiko Kimura, Yumi Kuninaka, Tamaki Yahata, Sakiko Nanjo, Saori Toujima, Sawako Minami, Kazuhiko Ino, Naofumi Mukaida, Toshikazu Kondo

**Affiliations:** 1 Department of Forensic Medicine, Wakayama Medical University, Wakayama, Japan; 2 Department of Obstetrics and Gynecology, Wakayama Medical University, Wakayama, Japan; 3 Division of Molecular Bioregulation, Cancer Research Institute, Kanazawa University, Kanazawa, Japan; Shanghai Jiao Tong University, CHINA

## Abstract

Preterm labor (PTL) is the most common cause of neonatal death and long-term adverse outcome. The pharmacological agents for PTL prevention are palliative and frequently fail to prevent PTL and improve neonatal outcome. It is essential to fully understand the molecular mechanisms of PTL in order to develop novel therapeutic methods against PTL. Several lines of evidence indicate some chemokines are expressed in gestational tissues during labor or PTL. To reveal the pathophysiological roles of the CX3CL1-CX3CR1 axis in PTL, we performed present study using LPS-induced PTL mice model in CX3CR1-deficient (*Cx3cr1*^*-/-*^) mice. We indicated that PTL was suppressed in *Cx3cr1*^*-/-*^ mice and immunoneutralization of CX3CL1 in WT mice. From immunohistochemical and the gene expression analyses, the CX3CL1-CX3CR1 axis has detrimental roles in PTL through intrauterine recruitment of macrophages and the enhancement of macrophage-derived inflammatory mediators. Thus, the CX3CL1-CX3CR1 axis may be a good molecular target for preventing PTL.

## Introduction

Preterm labor (PTL) is defined as labor arising from premature uterine contractility and occurs prior to 37 weeks of gestation in human. In the United States, approximately 11% of all births are diagnosed as preterm [1], and 15 million premature babies are estimated to be born annually worldwide as a result of PTL. PTL is the most common cause of neonatal death and can additionally cause long-term damages to the brain, bowel, lungs, and eyes, leading to severe lifetime handicap development [2]. As a consequence, PTL remains the biggest problem in obstetrics [3]. PTL prevention is attempted by the administration of pharmacological agents including adrenoreceptor agonists, cyclooxygenase inhibitors, magnesium sulphate, calcium-channel blockers and/or oxytocin antagonists in order to arrest or decrease premature uterine contractility. However, these drugs are palliative and frequently fail to prevent PTL and improve neonatal outcome [3].

It is essential to fully understand the molecular mechanisms of PTL in order to develop new therapeutic methods against it. Intra-amniotic infection has been causally linked to spontaneous PTL [4], as statistical analyses have estimated that infections are observed in 30% to 40% of all PTL cases [5]. Indeed, PTL ensues frequently from an increase in bacterial colonization, which originates as bacterial vaginosis and spreads over gestational tissues including choriodecidua, fetal membranes, amniotic cavity, and eventually the fetus.

PTL and term labor exhibit a common feature whereby an increase in inflammatory mediators, such as TNF-α, IL-1, IL-6 and IL-8, is observed in the amniotic fluid before the onset of uterine contraction. These mediators can induce the synthesis of prostaglandins (PGs), which are crucially involved in the expelling of fetus by robustly inducing myometrial contraction [6]. Thus, it is currently assumed that PTL is triggered by premature emergence of these pro-inflammatory mediators in gestational tissues [7].

Leukocytes, particularly neutrophils and macrophages, abundantly infiltrate the cervix, myometrium, and decidua during labor [8] and are a rich source of inflammatory mediators, thereby initiating and augmenting inflammatory reactions [9]. Leukocyte infiltration is mainly regulated by chemokines, which exhibit a potent chemotactic activity in leukocytes after binding to their specific G protein-coupled receptors with seven transmembrane portions. Chemokines are classified into four subgroups based on their cysteine motif: CXC, CC, CX3C, and C chemokines [10]. Gestational tissues at labor or PTL contain copiously several chemokines including IL-8, CCL2, CCL3, CCL5, CCL8, CCL18, CXCL1, CXCL6, CXCL9, and CXCL10 [11–15]. Their pathogenic roles were substantiated by the observation that a broad-spectrum chemokine inhibitor blocked the onset of labor in the infection-induced PTL mouse model [16].

CX3CL1/fractalkine is a unique member of CX3C family and is expressed by inflamed endothelial cells, macrophages, neurons, and glial cells [17]. CX3CL1 exists in both soluble and membrane-bound forms [18], and both forms bind to its single specific receptor, CX3CR1 [19], which is expressed monocyte/macrophage, lymphocytes, and NK cells. The CX3CL1-CX3CR1 axis was proposed to be crucially involved in inflammatory diseases such as rheumatoid arthritis, Crohn’s disease, atherosclerosis, allergic asthma, and sepsis [20–22]. Moreover, accumulating evidence has indicated the contribution of the CX3CL1-CX3CR1 axis to the pathogenesis of obstetric disorders including preeclampsia, gestational diabetes mellitus and miscarriage [23–25]. However, the still undetermined pathophysiological roles of the CX3CL1-CX3CR1 axis in PTL prompted us to explore it by using *Cx3cr1*-deficient (*Cx3cr1*^-/-^) mice.

In the present study, we demonstrated that the lack of CX3CR1 attenuated LPS-induced PTL, along with the reduction in intrauterine macrophage recruitment, inflammatory cytokine production, and expression of prostaglandin-endoperoxide synthase 2 (PTGS2), also known as COX-2. Thus, the CX3CL1-CX3CR1 axis may be a novel molecular target in the prevention of PTL.

## Materials and methods

### Reagents and antibodies (Abs)

LPS from *Escherichia coli* O55:B5 and recombinant mouse CX3CL1 were purchased from LIST Biological Laboratories (Campbell, CA) and R&D Systems (Minneapolis, MN), respectively. The following mAbs and polyclonal Abs (pAbs) were used for immunohistochemical or double-color immunofluorescence analyses; goat anti-CX3CL1/fractalkine pAbs (sc-7227), rabbit anti-IL-1β pAbs (sc-7884), rabbit anti-IL-6 pAbs (sc-1265-R), goat anti-TNF-α pAbs (sc-1350), goat anti-COX-2 pAbs (sc-1747) (all from Santa Cruz biotechnology, Santa Cruz, CA), rabbit anti-CX3CR1 pAbs (PAB16479, Abnova, Taipei, Taiwan), rabbit anti-myeloperoxidase (MPO) pAbs (RB-373-A, NeoMarkers, Fremont, CA), rat anti-mouse F4/80 mAb (MCA497G, AbD Serotec, Kidlington, UK), rabbit anti-human CD3 pAbs (A0452, Dako, Kyoto, Japan), rat anti-mouse pan-NK cells mAb (09441D(553855), BD, Franklin Lakes, NJ), rat anti-mouse F4/80 mAb (T-2028, BM8; eBioscience, San Diego, CA), cyanine dye 3-conjugated donkey anti-rat IgG pAbs (712-165-153), FITC-conjugated donkey anti-rabbit IgG (711-095-152, anti-goat IgG pAbs (705-095-147) (all from Jackson Immunoresearch Laboratories, West Grove, PA). The following Abs were used as neutralizing antibody; rat anti-mouse CX3CL1 mAb (MAB571), purified rat IgG (6-001-F) (all from R&D systems, Minneapolis, MN).

### Definition of clinical situations and sampling of blood and placentas from patients

Human sera and placentas were obtained at the Department of Obstetrics and Gynecology, Wakayama Medical University between 2011 and 2016. The clinical data of patients were extracted from medical records. As described previously [26], the study groups were defined as follows; deliver at term in which sera were obtained at the gestation of 28 to 32 weeks (preterm control), preterm with labor at the gestation of 28 to 32 weeks (PTL), and term with (TIL) or without (TNL) spontaneous labor. PTL group included threatened premature labor and preterm premature rupture of membrane (pPROM). Patients in PTL group were treated with antibiotics and tocolysis (ritodorine hydrochloride). Only when ritodorine hydrochloride was ineffective, magnesium sulfate (MgSO_4_) was additionally administered. Labor was defined by the presence of regular uterine contraction at a frequency of less than 10 min interval with cervical changes or rupture of membrane resulting in delivery [27]. In TIL and PTL groups, sera were obtained at the hospitalization. In TNL group, sera were collected before caesarian section. Patients who delivered neonates with congenital or chromosomal abnormalities, twin, and those with preeclampsia or gestational diabetes were excluded based on the clinical records. The demographic and clinical characteristics of each group are shown in Table 1. Placentas were obtained at the delivery, and several tissue sections consisting of the chorioamniotic membranes, umbilical cord, and placenta were evaluated for diagnosis of chorioamnionitis and funisitis, according to Blanc classification [28], by the pathologists without a prior knowledge. Human sera and placentas were obtained under the approval by the Institutional Review Boards of Wakayama Medical University.

**Table 1:** Demographics and clinical characteristics of serum samples used for comparison of serum CX3CL1 concentration.

	TNL	TIL	Preterm control	PTL	P value
**Gestational age at sampling**	38±0.11	39±0.25	29±0.21	28±0.55	<0.01
**Gestational age at delivery**	38±0.11	39±0.25	39±0.18	29±0.36	<0.01
**Maternal age**	34.3±0.81	32±1.17	30±0.98	29.5±11.24	0.018
**Assisted reproductive technique (%)**	10 (2/20)	5 (1/20)	0 (0/20)	5 (1/20)	0.96
**Multipara (%)**	75 (15/20)	75 (15/20)	60 (12/20)	70 (14/20)	0.83
**Smoking (%)**	0 (0/20)	0 (0/20)	0 (0/20)	5 (1/20)	0.99
**Medical history of PTL (%)**	0 (0/20)	0 (0/20)	0 (0/20)	5 (1/20)	0.99
**Caesarean section (%)**	100 (20/20)	5 (1/20)	10 (2/20)	25 (5/20)	<0.01
**Body weight of newborn (g)**	2941±82.7	3003±62.3	3024±76.0	1248±74.1	<0.01
**Apgar score at 1min**	0 (0/20)	0 (0/20)	0 (0/20)	35 (7/20)	<0.01
**Apgar score at 5min**	0 (0/20)	0 (0/20)	0 (0/20)	5 (1/20)	0.99
**Umbilical arterial pH**	5 (1/20)	0 (0/20)	0 (0/20)	0 (0/20)	0.99

(20 cases in each groups)

### Mice

Specific pathogen-free 8-12-week-old female C57BL/6 mice were purchased from SLC Japan Inc (Shizuoka, Japan). and designated as WT mice in this study. *Cx3cr1*^*-/-*^ mice were a generous gift from Drs. P.M. Murphy and J.L. Gao (National Institute of Allergy and Infectious Diseases, National Institutes of Health, Bethesda, MD) [29]. Age- and sex-matched *Cx3cr1*^*-/-*^ mice, which were backcrossed to C57BL/6 mice at least 8 generations, were used in the following experiments. All mice were housed individually in cages under specific pathogen-free conditions during the whole course of the study.

### Animal model

LPS-induced PTL was conducted similarly as described previously [16]. In brief, two female mice were co-housed with a male one of the same genotype and checked for vaginal plugs as the evidence of mating at the next morning. The day of vaginal plug detection was designated as gd 0.5 of pregnancy, and the pregnant mice were removed from the male. All pregnant mice delivered their pups on gd 19 or 20. At gd 15.5, the pregnant mice were intraperitoneally administered with LPS (25 μg in 200 μl of PBS) or PBS 200 μl, followed by the observation until delivery. The presence of intact or partial fetal tissue in the cage was noted as the evidence for delivery, and the delivery before gd 18.5 was judged as pre-term labor, and pups born before gd 18.5 could not survive >24 h after birth [30]. In our dose, 25 μg was the highest dose of LPS that reproducibly induced PTL without significant morbidity or mortality in gravid C57BL/6 females, based on the established method [31, 32]. In some experiments, pregnant WT mice were i.p. injected with anti-CX3CL1 Ab (200 μg/mouse) or control IgG at 3 h after LPS injection, and were observed until delivery. The percentage of preterm labor was calculated as follows: percentage (%) = 100 x (number of LPS-treated mice that delivered until gd 18.5/number of LPS-treated pregnant mice). All animal experiments were approved by the Committee on Animal Care and Use at Wakayama Medical University.

### Blood and tissue sampling

At gd 5.5, 10.5, 15.5, 18.5, or 6 h after LPS or PBS injection, mice were anesthetized by the intraperitoneal injection of Avertin (tribromoethanol, 20 μg/g), and whole blood samples were taken, followed by centrifugation (3,000 x *g* for 15 min) to collect serum. In each pregnant mouse, uterus was removed, and viable fetuses were dissected from the amniotic sac and umbilical cord, to weigh the obtained fetuses and placentas. Gestational tissues including uterus (from between implantation sites), placenta and amnion were stored at -80°C until processing for the subsequent analyses.

### Enzyme-linked immunosorbent assay (ELISA)

Human serum levels of CX3CL1/fractalkine, CCL2, CCL3, and CCL5, and mouse serum level of CX3CL1/fractalkine were measured with commercially available ELISA kit (R&D systems, Minneapolis, MN), according to the manufacturer’s recommendation. The detection limits; human CX3CL1/fractalkine, > 0.018 ng/ml; CCL2, > 1.7 pg/ml; CCL3, > 10 pg/ml; CCL5, > 2 pg/ml; and mouse CX3CL1/fractalkine > 0.21 ng/ml.

### Histopathological and immunohistochemical analyses

Human and mouse gestational tissues were fixed in 4% formaldehyde buffered with PBS (pH 7.2) and paraffin-embedded sections (4-μm thick) were made, followed by conventional H&E staining. Immunohistochemical analyses were performed as described previously [33]. Briefly, deparaffinized sections were immersed in 3% H_2_O_2_ in PBS for 5 min to eliminate endogenous peroxidase activity. The sections were further treated with PBS containing 1% normal serum corresponding to the secondary Abs and 1% BSA to reduce nonspecific reactions, and incubated with the primary Abs at the optimal conditions. After incubation with biotinylated secondary Abs, immune-complexes were visualized by Catalyzed Signal Amplification system (CSA) or Labelled Streptavidin biotin (LSAB) system (Dako, Kyoto, Japan), followed by counterstaining with hematoxylin. The positive cells were enumerated on 5 randomly-chosen visual fields at x400 magnifications, the total numbers in the five fields were combined. All measurements were carried out by two independent researchers without a prior knowledge of the experimental protocols.

### Double-color immunofluorescence analyses

A double-color immunofluorescence analysis was performed to identify the types of cells expressing CX3CR1-, IL-1β-, IL-6-, TNF-α-, or COX-2 in mouse tissues as described previously [34]. Deparaffinized sections were performed by enzymatic digestion with trypsin for 10 min at 37°C for antigen retrieval. The slides were incubated with PBS containing 1% normal donkey serum and 1% BSA to reduce nonspecific reactions. Thereafter, the slides were incubated overnight at 4°C with pairs of primary Abs. After washing, the slides were incubated with fluorochrome-conjugated secondary Abs at room temperature for 1 hr. The slides were then observed under a fluorescence microscopy.

### Cell culture

WT mice were i.p. injected with 2 ml of 4% thioglycolate (Sigma-Aldrich, St. Louis, MO), and intraperitoneal macrophages were harvested at 3 days later as described previously [29]. The resultant cell population consisted of more than 95% macrophages based on the morphological criteria. The cells were suspended in DMEM medium (containing 10% FBS and penicillin) and incubated at 37°C in six-well cell culture plates. Two h later, non-adherent cells were removed, and the medium was replaced. Thereafter, the cells were stimulated with CX3CL1 (1, 10 and 100 ng/ml) for 3 h and were subjected to subsequent analyses.

### Extraction of total RNAs and real-time RT-PCR

Total RNA was extracted from gestational tissue or peritoneal macrophages using ISOGEN II (Nippon Gene, Tokyo, Japan), according to the manufacturer’s instructions. One μg of total RNA was reverse transcribed into cDNA at 37°C for 15 min using PrimeScript RT reagent Kit with gDNA Eraser (Takara Bio, Shiga, Japan). Thereafter, generated cDNA was subjected to real-time PCR analysis using SYBR Premix Ex Taq II kit (Takara Bio, Shiga, Japan) with the specific primer sets (Table 2) obtained from Takara Bio. Amplification and detection of mRNA was performed using Thermal Cycler Dice Real Time System (TP800; Takara Bio, Shiga, Japan) according to the manufacturer’s instructions. Relative quantity of target gene expression to β-actin gene was measured by comparative Ct method as described previously [35].

**Table 2:** Sequences of the primers used for RT-PCR.

Transcript	Sequence
***Il1b***	(F) 5’-TCCAGGATGAGGACATGAGCAC-3’
	(R) 5’-GAACGTCACACACCAGCAGGTTA-3’
***Il6***	(F) 5’-CCACTTCACAAGTCGGAGGCTTA-3’
	(R) 5’-GCAAGTGCATCATCGTTGTTCATAC-3’
***Tnfa***	(F) 5’-AAGCCTGTAGCCCACGTCGTA-3’
	(R) 5’-GGCACCACTAGTTGGTTGTCTTTG-3’
***Ptgs2***	(F) 5’-GCCAGGCTGAACTTCGAAACA-3’
	(R) 5’-GCTCACGAGGCCACTGATACCTA-3’
***Actb***	(F) 5’-CATCCGTAAAGACCTCTATGCCAAC-3’
	(R) 5’-ATGGAGCCACCGATCCACA-3’

### Statistics

The means and SEMs were calculated and presented for all parameters determined in this study. Statistical significance was evaluated using, Mann-Whitney’s *U* test, Kruskal-Wallis test, Steel-Dwass test, or Chi-square test. *P*<0.05 was accepted as statistically significant.

## Results

### Elevated serum CX3CL1 levels in PTL patients

**Fig 1:**
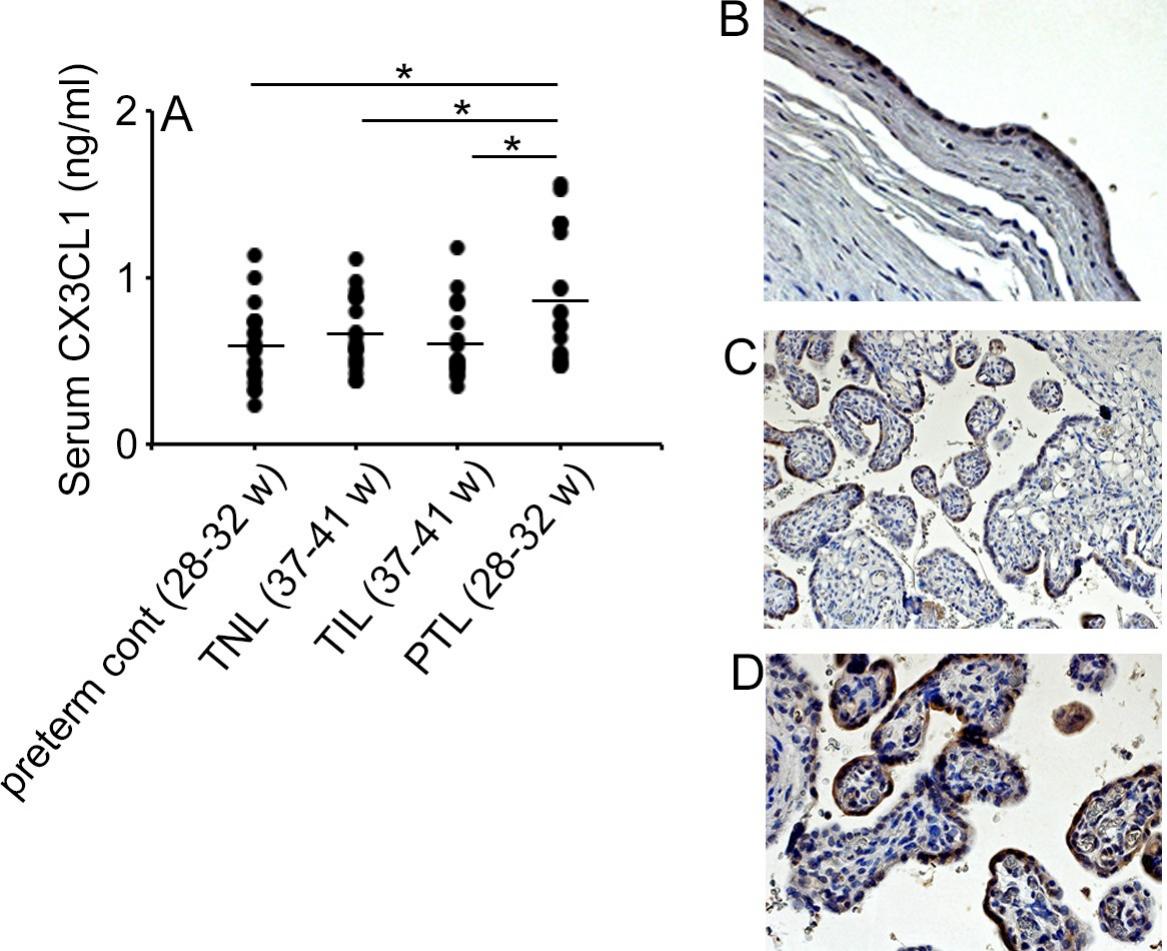
CX3CL1 expression in PTL of human. (A) Human serum CX3CL1 concentrations were determined on TNL, TIL, PTL and control groups as described in Materials and Methods. Statistical significance determined using Mann-Whitney’s *U* test, *P < 0.05 (n = 20 cases in each group). (B to D) Immunohistochemical detection of CX3CL1 protein in amnion epithelial cells (B) and syncytiotrophoblasts (C and D) of human placenta obtained from patients with grade III chorioamnionitis. Representative results from 6 individuals are shown here. Original magnification; B and C, x200; D, x400.

Serum CX3CL1 levels were not increased in term labor with spontaneous labor (TIL) and term labor without spontaneous labor (TNL), compared with preterm control group, indicating that labor itself cannot increase serum CX3CL1 levels (Fig 1A). On the contrary, the PTL group exhibited significantly elevated serum CX3CL1 level compared with those in the other groups (Fig 1A). CX3CL1 proteins were observed by immunohistochemistry in amnion epithelial cells and trophoblasts obtained from the placenta of patients who suffered from preterm labor arising from chorioamnionitis (Fig 1B–1D). On the contrary, chemokines, serum CCL2 and CCL5 levels were relatively but not significantly elevated in PTL group, compared to preterm control group (S1 Fig). Moreover, serum CCL3 levels could not be detected in all cases. These observations implied the involvement of CX3CL1 in the pathophysiology of PTL, particularly one that is associated with intrauterine infection.

### CX3CL1 and CX3CR1 expression in murine gestational tissues after LPS injection

**Fig 2:**
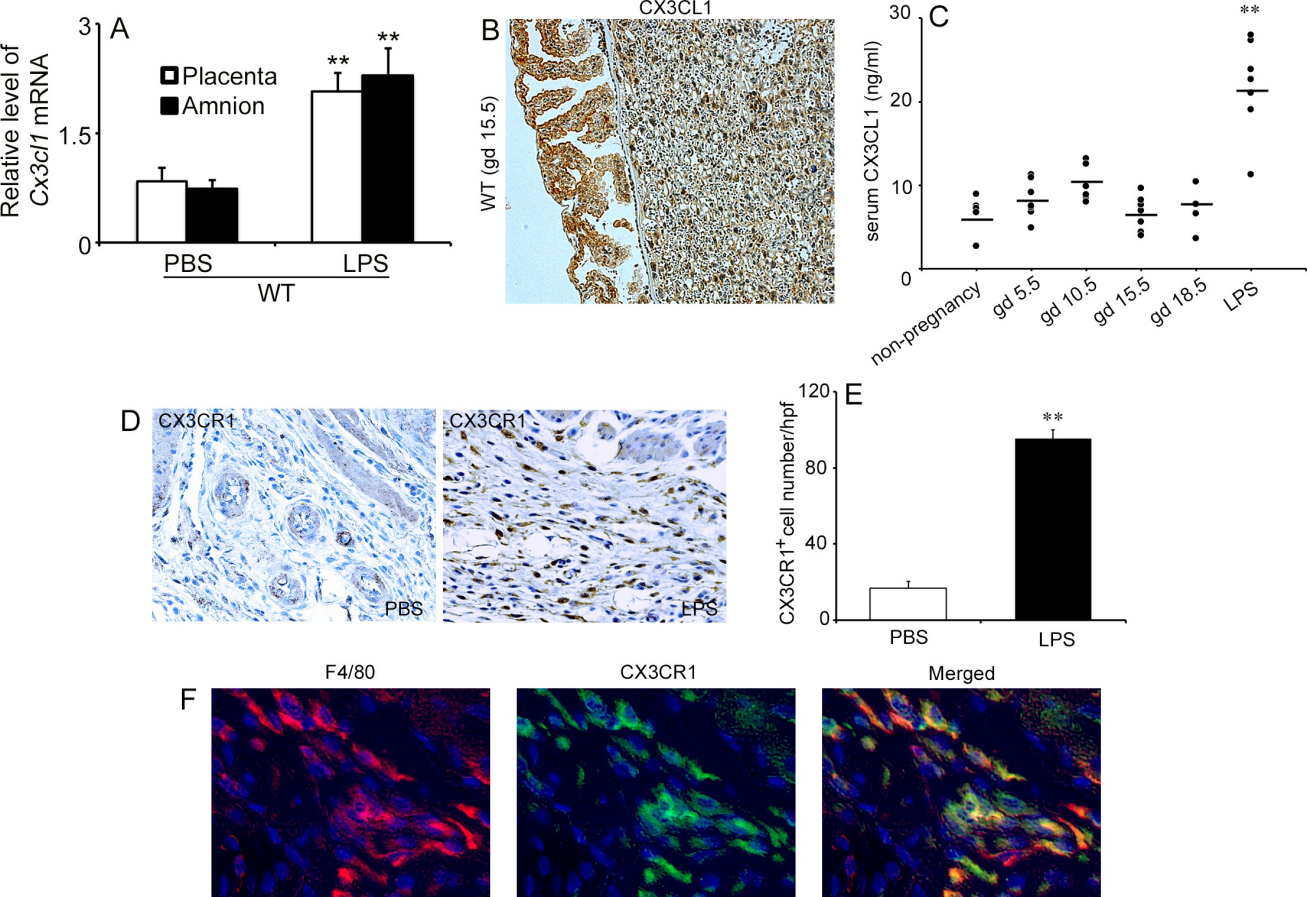
CX3CL1 and CX3CR1 expression in murine gestational tissues after LPS injection. (A) Gene expression of *Cx3cl1* in the gestational tissues (placenta and amnion) of pregnant WT mice at 6 h after LPS or PBS injection at gd 15.5. (B) Detection of CX3CL1 protein in murine gestational tissues. Immunohistochemical analysis was performed using anti-CX3CL1 Abs in WT mice at gd 15.5. Representative results from 6 independent animals are shown here. Original magnification, x200. (C) Serum CX3CL1 concentrations in non-pregnant and pregnant WT mice. The serum was collected at the indicated time points of pregnancy. Serum CX3CL1 levels were measured as described in Materials and Methods. Statistical significance determined using Mann-Whitney’s *U* test, **P < 0.01, vs. non-pregnancy and other gestational ages (n = 5–8 cases in each group). (D) Immunohistochemical detection of CX3CR1 in the uterus of pregnant WT mice at 6 h after PBS or LPS injection. Representative results from 6 independent experiments are shown here. Original magnification, x400. (E) Enumeration of CX3CR1-positive cells in the gestational tissues of pregnant WT mice after PBS or LPS treatment. All values represent the mean ±SEM. Statistical significance determined using Mann-Whitney’s *U* test, **P < 0.01, vs. PBS treatment (n = 5–8 cases in each group). (F) Double-color immunofluorescence analysis for the identification of CX3CR1-expressing cells in the uterus of pregnant WT mice at 6 h after LPS injection. Representative results from six independent experiments are shown here. Signals were digitally merged (F4/80 and CX3CR1). Original magnification, ×400.

LPS treatment enhanced *Cx3cl1* gene expression in the gestational tissues of pregnant WT mice at gestational day (gd) 15.5 (Fig 2A). Consistently, immunohistochemical analysis detected CX3CL1 proteins in amnion epithelial cells and cytotrophoblasts within the labyrinth zone in the placenta of pregnant WT mice at gd 15.5 (Fig 2B). In contrast, serum CX3CL1 levels fluctuated but did not change significantly until gd 18.5 compared with that in non-pregnant mice (Fig 2C). LPS injection at gd 15.5, however, markedly increased serum CX3CL1 levels, at 6 h after injection (Fig 2C) and induced preterm labor in most WT animals (7 cases of PTL in 8 LPS-treated WT mice). Moreover, a large number of CX3CR1-expressing cells appeared in uterus at 6 h after LPS injection compared with PBS treatment (Fig 2D and 2E). Furthermore, a double-color immunofluorescence analysis identified most CX3CR1-expressing cells in the uterus as F4/80-positive macrophages (Fig 2F). These observations implied that LPS-induced PTL was accompanied by the infiltration of CX3CR1-expressing macrophages into the uterus.

### Crucial roles of the CX3CL1-CX3CR1 axis in LPS-induced PTL

**Fig 3:**
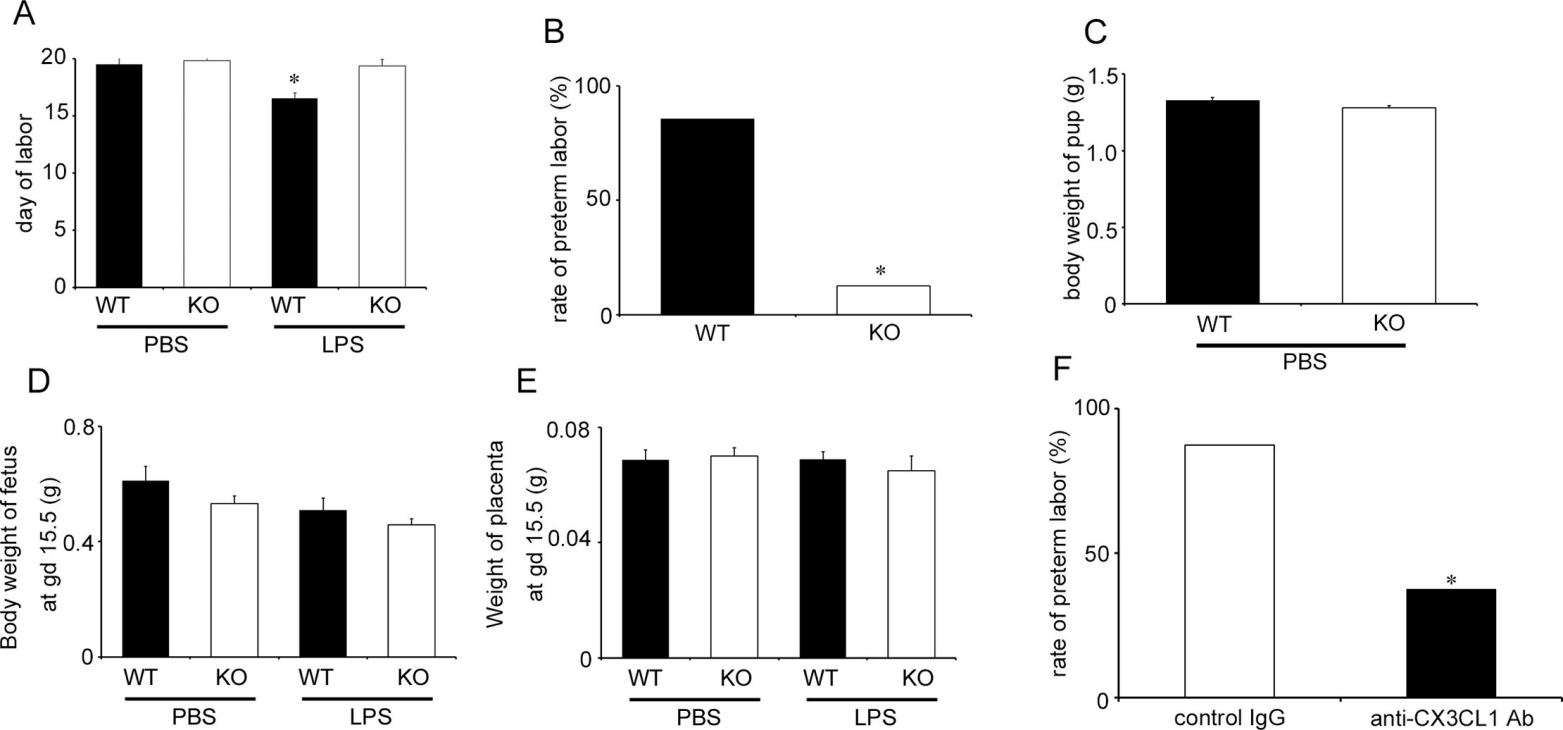
The roles of the CXCL1-CX3CR1 axis in LPS-induced PTL. (A) Duration of pregnancy in WT and Cx3cr1^-/-^ (KO) mice when PBS or LPS (25 μg/mouse) was injected intraperitoneally at gd 15.5. Statistical significance determined using Kruskal-Wallis test and Steel-Dwass test, *P < 0.05; vs. WT mice treated with PBS (n = 5–8 cases in each group). (B) The rate of PTL in WT and KO mice treated with LPS (25 μg/mouse) at gd 15.5. Statistical significance determined using Mann-Whitney’s *U* test, *P < 0.05; vs. WT mice (n = 5–8 cases in each group). (C) Body weights of pups delivered by pregnant WT and KO mice after PBS injection at gd 15.5. The Mann-Whitney’s *U* test was used (n = 5 cases in each group). (D) Body weights of fetus removed from pregnant mice at 6 h after PBS or LPS (25 μg/mouse) injection at gd 15.5. The Kruskal-Wallis test was used (n = 5–8 cases in each group). (E) Weights of placenta obtained from pregnant mice at 6 h after PBS or LPS (25 μg/mouse) injection at gd 15.5. The Kruskal-Wallis test was used (n = 5–8 cases in each group). (F) The effects of anti-CX3CL1 Abs on PTL of pregnant WT mice with LPS injection. WT mice were treated with anti-CX3CL1 Abs or control IgG at 3 h after LPS (25 μg/mouse) injection at gd 15.5. Statistical significance determined using Mann-Whitney’s *U* test. *P < 0.05, vs. mice treated with control IgG (n = 8 cases in each group).

In order to evaluate the roles of the CX3CL1-CX3CR1 axis in PTL, we injected WT and *Cx3cr1*^*-/-*^ mice with LPS since CX3CR1 is a single specific receptor for CX3CL1. WT and *Cx3cr1*^*-/-*^ mice went through labored at term either without treatment (S2 Fig) or with PBS treatment (Fig 3A). On the contrary, LPS injection markedly reduced pregnancy duration and caused PTL in most WT (6 out of 7, 85.7%) but a minority of *Cx3cr1*^*-/-*^ (1 out of 8 mice, 12.5%) mice (Fig 3A and 3B). WT and *Cx3cr1*^*-/-*^ mice delivered pups with the same body weights when they delivered spontaneously at full-term (Fig 3C). Moreover, LPS administration at gd 15.5 had few impacts on the fetal body and placenta weights in both WT and *Cx3cr1*^*-/-*^ mice until 6 h after the injection (Fig 3D and 3E). In order to exclude the possibility that genetic deficiency of CX3CR1 may cause secondary effects on labor processes, we next examined the effects of anti-CX3CL1 on LPS-induced PTL, since CX3CL1 is the sole ligand for CX3CR1. Anti-CX3CL1 injection significantly reduced LPS-induced PTL in WT mice compared with that in control IgG treatment (anti-CX3CL1, 3 out of 8 mice, 37.5%; control IgG, 7 out of 8 mice, 87.5%) (Fig 3F). These observations implicated the crucial involvement of the CX3CL1-CX3CR1 axis in LPS-induced PTL.

### Reduced intrauterine macrophage recruitment in pregnant *Cx3cr1*^-/-^ mice treated with LPS

**Fig 4:**
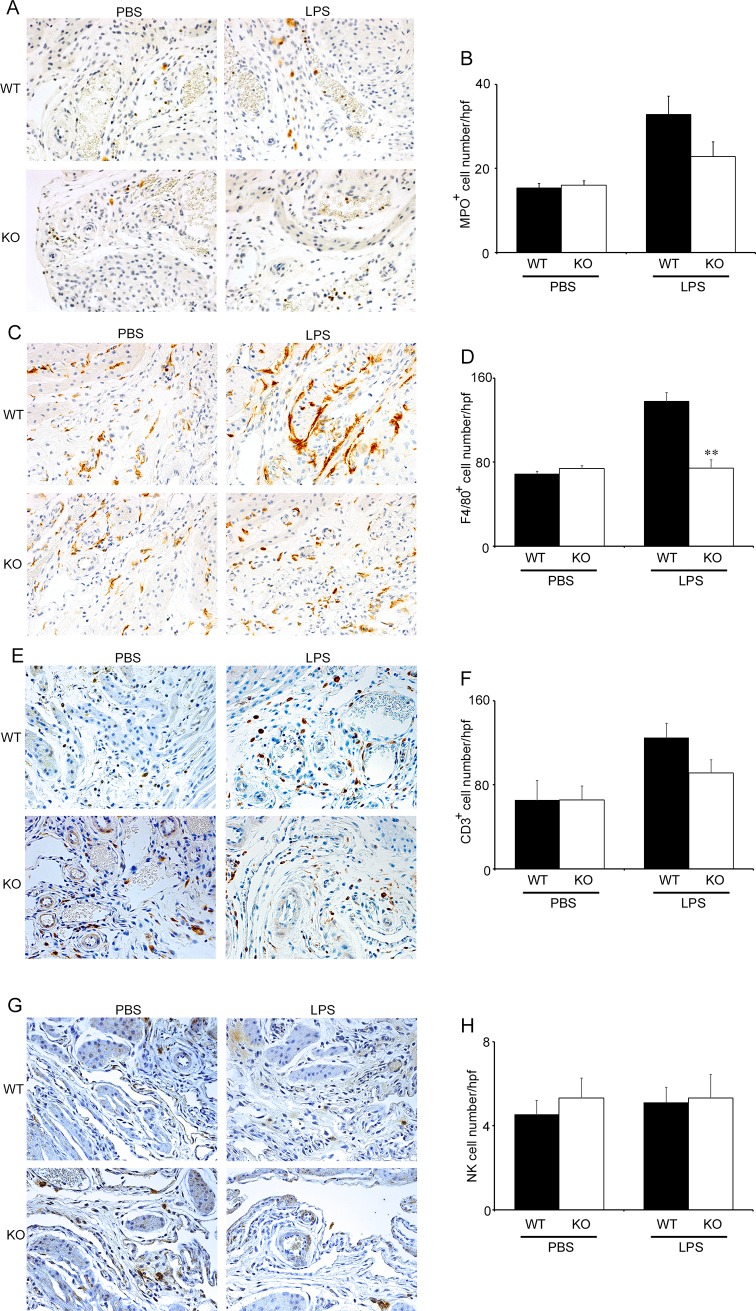
The comparison of intrauterine leukocyte recruitment between WT and *Cx3cr1*^*-/-*^ mice. Evaluation of intrauterine leukocyte infiltration in WT and KO mice at 6 h after PBS or LPS injection (25 μg/mouse) at gd 15.5. Immunohistochemical analysis was performed using anti-MPO pAbs (A and B), anti-F4/80 mAb (C and D), anti-CD3 pAbs (E and F), and anti-pan NK cell mAb (G and H) as described in Materials and Methods. Representative results from 6 independent experiments are shown in panels A, C, E, and G. Original magnification, ×400. Enumeration of each leukocyte population including neutrophils (B), macrophages (D), T cells (F), and NK cells (H) in WT and KO mice at 6 h after PBS or LPS (25 μg/mouse) injection at gd 15.5. All values represent the mean ± SEM; Statistical significance determined using Mann-Whitney’s *U* test. **P < 0.01 vs. WT (n = 5–8 cases in each group).

Since neutrophils and macrophages infiltrated the decidua during labor [36], we next explored leukocyte infiltration in pregnant WT and *Cx3cr1*^*-/-*^ mice during the course of LPS-induced PTL. Indeed, neutrophils, macrophages, T cells, and NK cells infiltrated into decidua at gd 15.5 to similar extents in both pregnant WT and *Cx3cr1*^*-/-*^ mice without LPS treatment (S3 Fig). LPS enhanced the infiltration of neutrophils, macrophages and T cells but not NK cells in WT mice at 6 h after the injection, compared with PBS treatment (Fig 4A–4H). Consistent with CX3CR1 expression on F4/80^+^ macrophages (Fig 2E), macrophage infiltration was not enhanced in *Cx3cr1*^*-/-*^ mice after LPS treatment (Fig 4C and 4D). On the contrary, neutrophil and T cell infiltration was not significantly suppressed in *Cx3cr1*^*-/-*^ mice (Fig 4A, 4B, 4E and 4F). These observations implicated that CX3CR1 deficiency may prevent LPS-induced PTL by suppressing intrauterine macrophage recruitment.

### Intrauterine gene expression of inflammatory mediators

**Fig 5:**
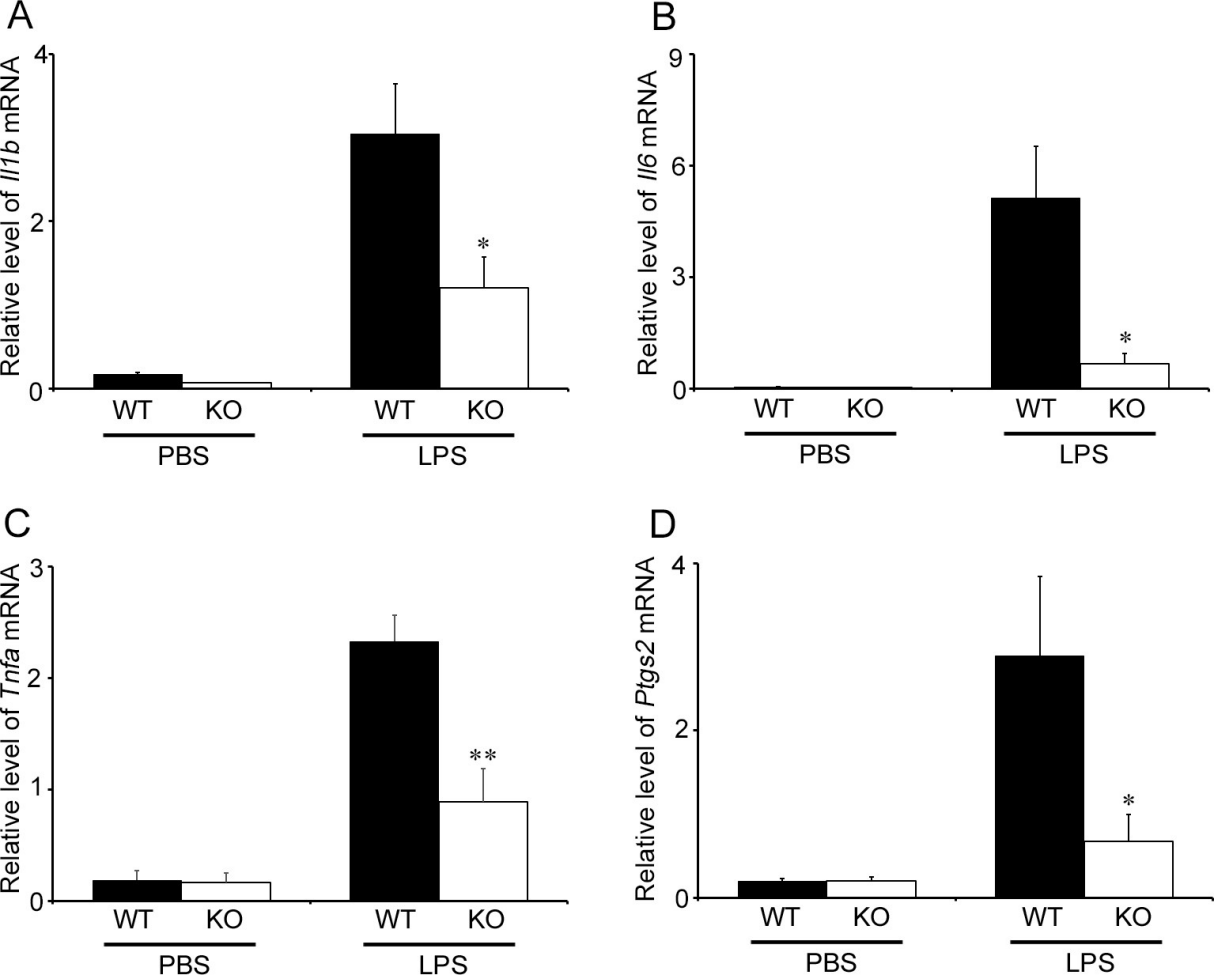
Intrauterine gene expression of inflammatory mediators in WT and *Cx3cr1*^*-/-*^ mice. The intrauterine gene expression of *Il1b* (A), *Il6* (B), *Tnfa* (C), and *Ptgs2* (D) in WT and KO mice uterus at 6 h after LPS (25 μg/mouse) or PBS injection at gd 15.5. Quantitative RT-PCR analyses were performed as described in Materials and Methods. All values represent the mean ± SEM; Statistical significance determined using Mann-Whitney’s *U* test. *P < 0.05, **P < 0.01 vs. WT (n = 5–8 cases in each group).

**Fig 6:**
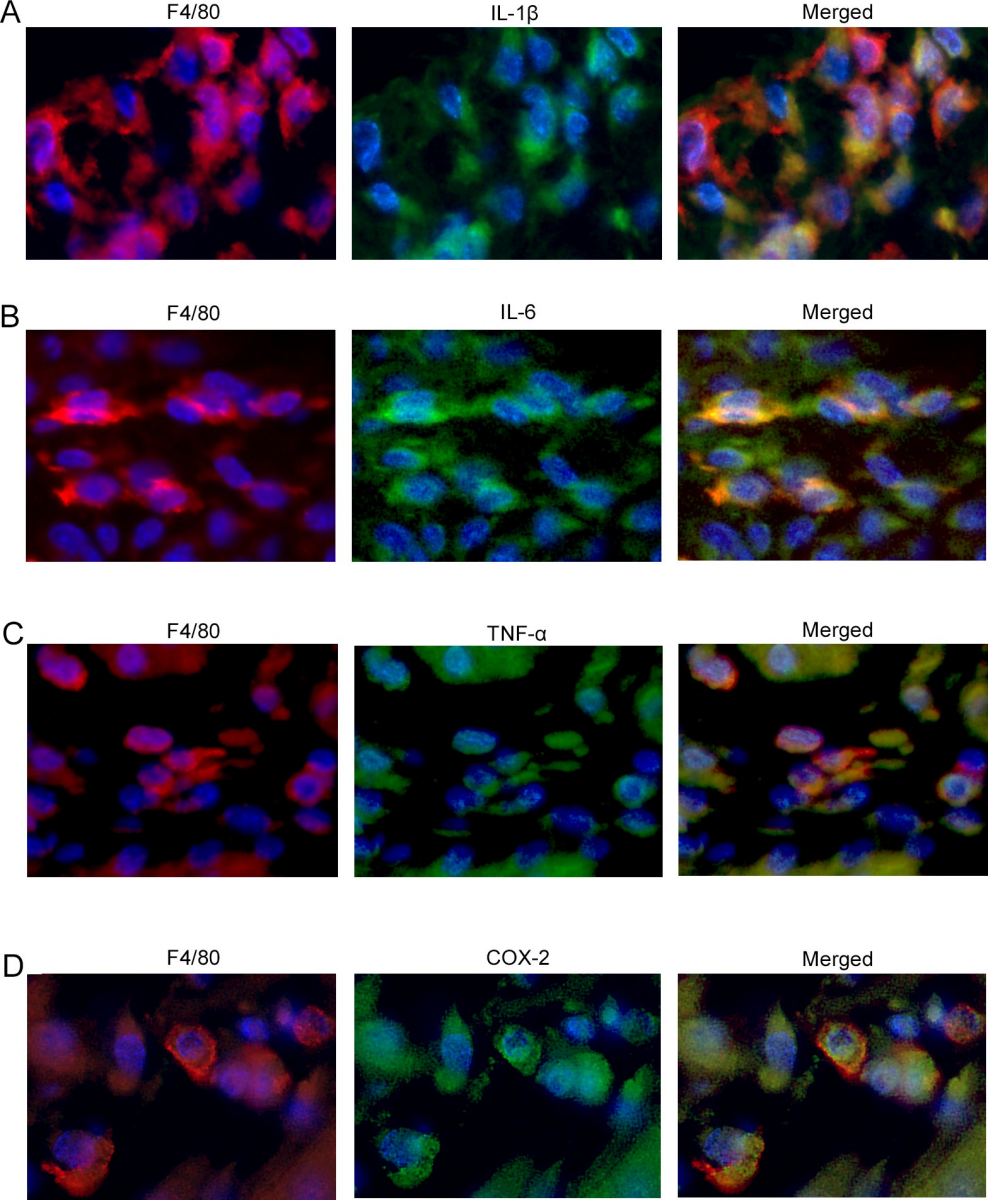
The expression of inflammatory mediators in F4/80^+^ macrophages infiltrating into the uterus. A double-color immunofluorescence analysis to identify the cells expressing inflammatory cytokines and COX-2 in the uterus of pregnant WT mice at 6 h after LPS (25 μg/mouse) injection at gd 15.5. Signals were merged digitally in the right panel of each row (A, F4/80 and IL-1β; B, F4/80 and IL-6; C, F4/80 and TNF-α; D, F4/80 and COX-2). Representative results from 6 independent experiments are shown here. Original magnification, ×400.

**Fig 7:**
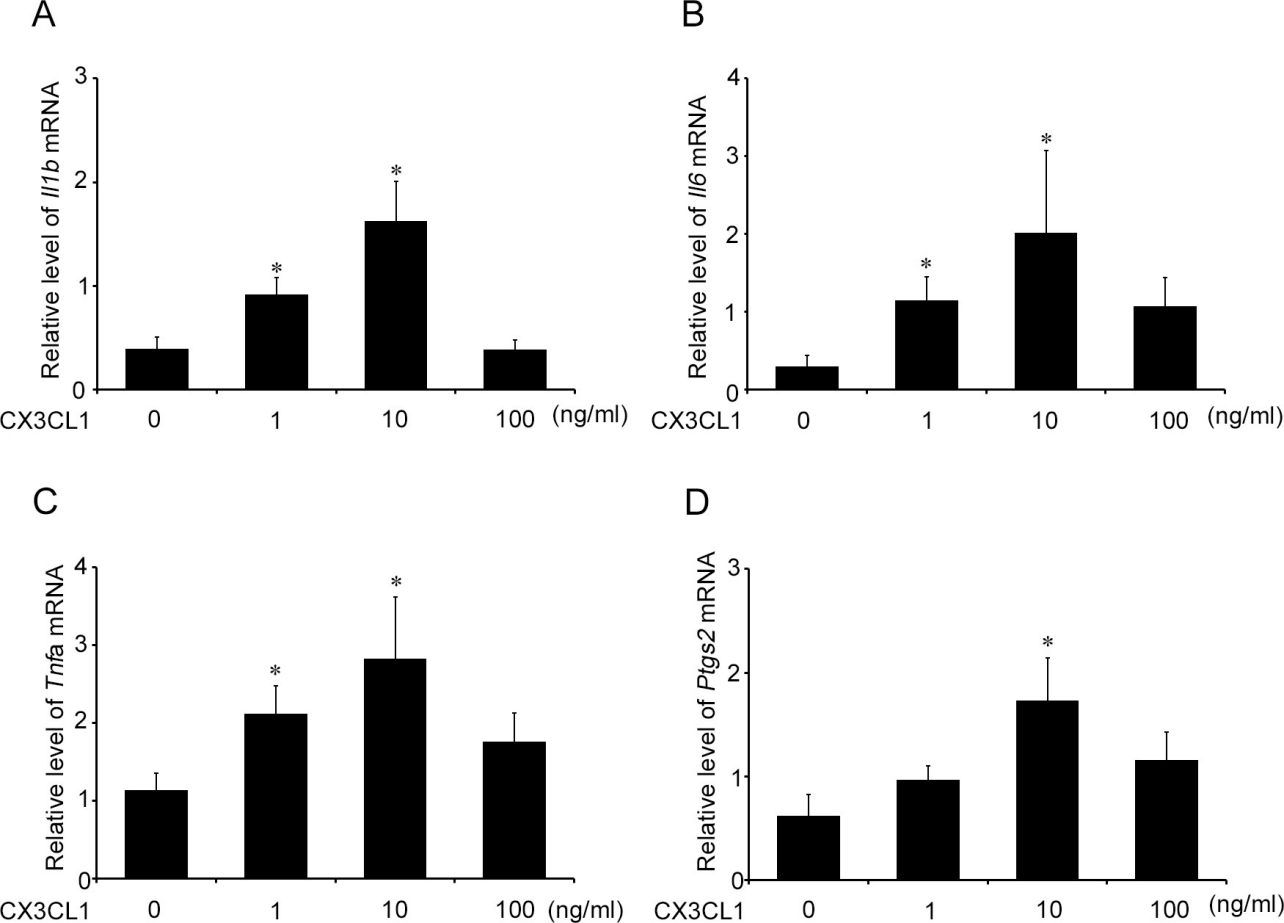
The effects of CX3CL1 on the gene expression of inflammatory mediators in WT mice-derived peritoneal macrophages. WT mouse-derived peritoneal macrophages were incubated with the indicated concentrations of CX3CL1 for 3 h. RT-PCR was performed on the extracted total RNAs as described in Materials and Methods. The gene expression of *Il1b* (A), *Il6* (B), *Tnfa* (C) *and Ptgs2* (D) are shown. All values represent the mean ± SEM; Statistical significance determined using Mann-Whitney’s *U* test. *, *P* < 0.05; vs. no CX3CL1 treatment (n = 10 cases in each group).

Accumulating evidence has indicated the vital roles of pro-inflammatory cytokines including IL-1β, IL-6, and TNF-α, and prostaglandin E_2_ in LPS-induced PTL [37]. Hence, we examined the intrauterine gene expression of *Il1b*, *Il6*, *Tnfa*, *and Ptgs2*. Both WT and *Cx3cr1*^*-/-*^ mice exhibited similar levels of intrauterine mRNA expression of these molecules at 6 h after PBS treatment at gd 15.5 (Fig 5). LPS treatment markedly enhanced intrauterine gene expression of these molecules in both WT and *Cx3cr1*^*-/-*^ mice, but the enhancement was significantly attenuated in *Cx3cr1*^*-/-*^ mice compared with that in WT ones (Fig 5). Moreover, a double-color immunofluorescence analyses revealed that IL-1β, IL-6, TNF-α, and COX-2 proteins were expressed mostly in F4/80^+^ macrophages, which infiltrated into the uterus at 6 h after LPS injection (Fig 6). Furthermore, CX3CL1 treatment augmented the gene expression of these molecules in WT mice-derived peritoneal macrophages in a dose-dependent manner (Fig 7). These observations indicated that CX3CL1 induced the infiltration of CX3CR1-expressing macrophages in the uterus and the simultaneous expression of these pro-inflammatory molecules, thereby promoting PTL development.

## Discussion

PTL, a major obstetrical complication, is associated commonly with systemic and/or local bacterial infection during pregnancy. Various types of cells including inflamed endothelial cells, macrophages, neurons, and glial cells express CX3CL1 [17], which exerts its activity by binding to its specific receptor, CX3CR1 [19]. We previously provided definitive evidence to indicate the crucial involvement of the CX3CL1-CX3CR1 in several bacterial infection models [29, 38]. Moreover, in line with previous reports [39, 40, 41], we could find that CX3CL1 was immunohistochemically expressed in amnion epithelial cells and trophoblasts in both human and murine samples. Hence, we examined the pathogenic roles of CX3CL1 in PTL. Indeed, in the present clinical and experimental studies, elevated serum CX3CL1 levels were related to PTL. CX3CR1-expressing cells markedly infiltrated the gestation tissues in mouse PTL model and genetic disruption of CX3CR1 gene or anti-CX3CL1 antibody administration prevented LPS-induced PTL in mice. Collectively, the CX3CL1-CX3CR1 axis can have important pathophysiological roles in infection-induced PTL.

Prior to delivery arising from either term labor or PTL, gestational tissues exhibit inflammatory responses [36], which induces uterine contraction, an indispensable step for labor [8, 42]. Inflammatory responses are associated with infiltration of various type of leukocytes including neutrophils, NK cells and macrophages. However, neutrophil recruitment had no influence on LPS-induced PTL [43]. The involvement of NK cells was proposed as evidenced by NK cell dysregulation in preterm labor in human [44] and NK cell depletion mediated delayed term in mice [45]. This assumption, however, could not be applied to the present model as evidenced by the absence of differences in intrauterine NK cells between WT and *Cx3cr1*^*-/-*^ mice. Macrophages consistently reside in mouse decidual tissues but are increased as gestational age advances, reaching the maximal level at gd 18, immediately before term labor. Indeed, the systemic depletion of macrophages could significantly prevent LPS-induced PTL in mice [37]. In the present study, macrophage recruitment was significantly suppressed together with PTL prevention in *Cx3cr1*^*-/-*^ mice compared with WT mice. Thus, macrophage infiltration may be associated with LPS-induced PTL.

Like other types of leukocytes, macrophage recruitment is also regulated under the guidance of chemokines systems [46]. Indeed, F4/80^+^ macrophages express various chemokine receptors including CX3CR1, and genetic disruption or immunoneutralization of CX3CR1 impaired macrophage recruitment in atherosclerosis, wound healing, and renal diseases, thereby preventing or alleviating pathological changes [47–51]. On the contrary, the absence of CX3CR1 gene had few effects on macrophage infiltration in other disease models such as thioglycolate-induced or cecal ligation and puncture-induced peritonitis [29, 52, 53]. Thus, CX3CR1-mediated signals may have distinct roles in macrophage infiltration in a context-dependent manner. In the present study, *Cx3cr1*^*-/-*^ mice showed impaired macrophage infiltration after LPS treatment, indicating the essential involvement of the CX3CL1-CX3CR1 interaction in macrophage recruitment in this PTL model.

Before delivery, the expression of several pro-inflammatory mediators is increased in the gestational tissues, together with concomitant intrauterine leukocyte influx [6, 54, 55]. Indeed, the expression of several pro-inflammatory cytokines is increased in pregnancies complications caused by infection or PTL in humans and other species [56–58]. These observations suggest the essential involvement of these mediators in PTL. In support of this notion, IL-1 and TNF-α administration induced both preterm labor and intermediate steps in the labor cascade, and genetic disruption of IL-1 or TNF-α receptors significantly prevented LPS-induced PTL [59–61]. In addition, the inhibition of IL-6 signaling or IL-6 production suppressed LPS-induced PTL [30, 62]. On the contrary, the absence of IL-10, a major anti-inflammatory cytokine, exaggerated LPS-induced PTL with elevated expression of inflammatory cytokines such as IL-1, IL-6 and TNF-α [63]. Thus, proinflammatory cytokines and IL-10 can promote and alleviate PTL, respectively. In the present study, PTL was alleviated in pregnant *Cx3cr1*^*-/-*^ mice, while intrauterine gene expression levels of *Il1b*, *Il6* and *Tnfa* were attenuated. Moreover, IL-1β, IL-6, and TNF-α were expressed in CX3CR1-expressing F4/80^+^ macrophages, and CX3CL1 enhanced their expression. Thus, CX3CR1-mediated signals could promote PTL, partly by inducing macrophage infiltration and their pro-inflammatory cytokine expression.

In both term labor and PTL, PGs play essential roles in the processes of uterine contractility, membrane rupture, and cervical ripening [64–66]. PGs are generated by the enzymatic action of PTGS, known as COX. PTGS1 (COX1) is constitutively expressed in most cell types, whereas PTGS2 (COX2) is induced in response to inflammatory stimuli [67]. The expression of *PTGS2* but not *PTGS1* is increased at term labor in human gestational tissues such as amnion, choriodecidua and myometrium [68, 69]. The administration of PTGS2 (COX-2) inhibitor can effectively suppress LPS-induced PTL [70, 71], implying the crucial roles of COX-2-mediated PGs. We proved that the lack of CX3CR1 decreased the intrauterine gene expression of *Ptgs2* after LPS treatment. In vitro, CX3CL1 treatment augmented the gene expression of *Ptgs2*, in addition to that of Il1b, Il6, and Tnfa, in WT-derived peritoneal macrophages in a dose-dependent manner. Because of the cytotoxicity, the highest dose of CX3CL1 at 100 ng/ml had no effects on the expression of these molecules in vitro. Moreover, a double-color immunofluorescence analysis detected COX-2 in F4/80^+^ macrophages, and CX3CL1 induced COX-2 expression in macrophages. Thus, the CX3CL1-CX3CR1 interaction prematurely induced COX-2-expressing macrophage infiltration and further enhanced COX-2 expression in macrophages, thereby inducing PTL.

Currently, uterine relaxant or tocolytic drugs are mainly employed to prevent PTL [2]. However, after systemic and/or local infection advances to inflammatory cascades, these drugs cannot effectively prevent PTL. Thus, it is necessary to develop more specific and effective therapeutic agents against infection-induced PTL. The blockade of CX3CL1-CX3CR1 axis significantly inhibited inflammation-induced PTL but did not result in any adverse effects on uninfected and uncomplicated labor. Thus, CX3CL1-CX3CR1 axis may be a novel molecular target for the development of therapies that prevent infection-related PTL.

## Supporting information

S1 Fig The chemokine levels of human serum. Human serum concentrations were determined on TNL, TIL, preterm control, and PTL groups (n = 20 in each group). (A) The serum CCL2 concentrations. (B) The serum CCL3 was not detected (n.d.) in all cases. (C) The serum CCL5 concentrations. Statistical significance determined using Mann-Whitney’s *U* test. *P < 0.05.
(TIF)

S2 Fig Duration of pregnancy in WT and Cx3cr1^-/-^ mice with untreatment. WT and *Cx3cr1*^-/-^ mice labored at term when they were untreated (n = 5–8 in each group).
(TIF)

S3 Fig The comparison of intrauterine leukocyte recruitment between untreated-WT and -*Cx3cr1*^*-/-*^ mice. Evaluation of intrauterine leukocyte infiltration in WT and *Cx3cr1*^*-/-*^ mice at gd 15.5 when they were untreated. Immunohistochemical analysis was performed using anti-MPO pAbs (A and B), anti-F4/80 mAb (C and D), anti-CD3 pAbs (E and F), and anti-pan NK cell mAb (G and H) as described in Materials and Methods. Representative results from 6 independent experiments are shown in panels A, C, E, and G. Original magnification, ×400. Enumeration of each leukocyte population including neutrophils (B), macrophages (D), T cells (F), and NK cells (H) in WT and *Cx3cr1*^*-/-*^ mice with untreatment at gd 15.5. All values represent the mean ± SEM (n = 5–8 in each group).
(TIF)

## References

[1] Martin JA, Hamilton BE, Osterman MJ, Curtin SC, Matthews TJ. Births: final data for 2013. Natl Vital Stat Rep. 2015;64(1):1–65. 25603115

[2] Walker KF, Thornton JG. Tocolysis and preterm labour. Lancet. 2016;387(10033):2068–2070. 10.1016/S0140-6736(16)00590-0 26944025

[3] Lawn JE, Kinney MV, Belizan JM, Mason EM, McDougall L, Larson J, et al Born Too Soon Preterm Birth Action Group. Born too soon: accelerating actions for prevention and care of 15 million newborns born too soon. Reprod Health. 2013;10 Suppl 1:S6 10.1186/1742-4755-10-S1-S6 24625252 PMC3828574

[4] Romero R, Gómez R, Chaiworapongsa T, Conoscenti G, Kim JC, Kim YM. The role of infection in preterm labour and delivery. Paediatr Perinat Epidemiol. 2001;15 Suppl 2:41–56. 11520399 10.1046/j.1365-3016.2001.00007.x

[5] Haram K, Mortensen JH, Wollen AL. Preterm delivery: an overview. Acta Obstet Gynecol Scand. 2003;82(8):687–704. 12848639 10.1034/j.1600-0412.2003.00218.x

[6] Mohan AR, Loudon JA, Bennett PR. Molecular and biochemical mechanisms of preterm labour. Semin Fetal Neonatal Med. 2004;9(6):437–44. 10.1016/j.siny.2004.08.001 15691781

[7] Romero R, Dey SK, Fisher SJ. Preterm labor: one syndrome, many causes. Science. 2014;345(6198):760–5. 10.1126/science.1251816 25124429 PMC4191866

[8] Hamilton S, Oomomian Y, Stephen G, Shynlova O, Tower CL, Garrod A, et al Macrophages infiltrate the human and rat decidua during term and preterm labor: evidence that decidual inflammation precedes labor. Biol Reprod. 2012;86(2):39 10.1095/biolreprod.111.095505 22011391

[9] Tower CL, Lui S, Jones RL. Are chemokines therapeutic targets for the prevention of preterm labor? Immunotherapy. 2014;6(8):901–4. 10.2217/imt.14.70 25313568

[10] Mukaida N. Pathophysiological roles of interleukin-8/CXCL8 in pulmonary diseases. Am J Physiol Lung Cell Mol Physiol. 2003;284(4):L566–77. 10.1152/ajplung.00233.2002 12618418

[11] Haddad R, Tromp G, Kuivaniemi H, Chaiworapongsa T, Kim YM, Mazor M, et al Human spontaneous labor without histologic chorioamnionitis is characterized by an acute inflammation gene expression signature. Am J Obstet Gynecol. 2006;195(2):394.e1–24. 16890549 10.1016/j.ajog.2005.08.057 PMC1800883

[12] Shynlova O, Lee YH, Srikhajon K, Lye SJ. Physiologic uterine inflammation and labor onset: integration of endocrine and mechanical signals. Reprod Sci. 2013;20(2):154–67. 10.1177/1933719112446084 22614625

[13] Mittal P, Romero R, Tarca AL, Gonzalez J, Draghici S, Xu Y, et al Characterization of the myometrial transcriptome and biological pathways of spontaneous human labor at term. J Perinat Med. 2010;38(6):617–43. 10.1515/JPM.2010.097 20629487 PMC3097097

[14] Hassan SS, Romero R, Haddad R, Hendler I, Khalek N, Tromp G, et al The transcriptome of the uterine cervix before and after spontaneous term parturition. Am J Obstet Gynecol. 2006;195(3):778–86. 10.1016/j.ajog.2006.06.021 16949412

[15] Hamilton SA, Tower CL, Jones RL. Identification of chemokines associated with the recruitment of decidual leukocytes in human labour: potential novel targets for preterm labour. PLoS One. 2013;8(2):e56946 10.1371/journal.pone.0056946 23451115 PMC3579936

[16] Shynlova O, Dorogin A, Li Y, Lye S. Inhibition of infection-mediated preterm birth by administration of broad spectrum chemokine inhibitor in mice. J Cell Mol Med. 2014;18(9):1816–29. 10.1111/jcmm.12307 24894878 PMC4196657

[17] Fong AM, Robinson LA, Steeber DA, Tedder TF, Yoshie O, Imai T, et al Fractalkine and CX3CR1 mediate a novel mechanism of leukocyte capture, firm adhesion, and activation under physiologic flow. J Exp Med. 1998;188(8):1413–9. 9782118 10.1084/jem.188.8.1413 PMC2213407

[18] Bazan JF, Bacon KB, Hardiman G, Wang W, Soo K, Rossi D, et al A new class of membrane-bound chemokine with a CX3C motif. Nature. 1997;385(6617):640–4. 10.1038/385640a0 9024663

[19] Imai T, Hieshima K, Haskell C, Baba M, Nagira M, Nishimura M, et al Identification and molecular characterization of fractalkine receptor CX3CR1, which mediates both leukocyte migration and adhesion. Cell. 1997;91(4):521–30. 9390561 10.1016/s0092-8674(00)80438-9

[20] Liu W, Jiang L, Bian C, Liang Y, Xing R, Yishakea M, et al Role of CX3CL1 in Diseases. Arch Immunol Ther Exp (Warsz). 2016;64(5):371–83. 10.1007/s00005-016-0395-9 27098399

[21] Julia V. CX3CL1 in allergic diseases: not just a chemotactic molecule. Allergy. 2012;67(9):1106–10. 10.1111/j.1398-9995.2012.02870.x 22765026

[22] Nishimura M, Kuboi Y, Muramoto K, Kawano T, Imai T. Chemokines as novel therapeutic targets for inflammatory bowel disease. Ann N Y Acad Sci. 2009;1173:350–6. 10.1111/j.1749-6632.2009.04738.x 19758172

[23] Siwetz M, Dieber-Rotheneder M, Cervar-Zivkovic M, Kummer D, Kremshofer J, Weiss G, et al Placental fractalkine is up-regulated in severe early-onset preeclampsia. Am J Pathol. 2015;185(5):1334–43. 10.1016/j.ajpath.2015.01.019 25769431 PMC4486762

[24] Ebert T, Hindricks J, Kralisch S, Lossner U, Jessnitzer B, Richter J, et al Serum levels of fractalkine are associated with markers of insulin resistance in gestational diabetes. Diabet Med. 2014;31(8):1014–7. 10.1111/dme.12451 24673545

[25] Li ZY, Chao HH, Liu HY, Song ZH, Li LL, Zhang YJ, et al IFN-γ induces aberrant CD49b⁺ NK cell recruitment through regulating CX3CL1: a novel mechanism by which IFN-γ provokes pregnancy failure. Cell Death Dis. 2014;5:e1512 10.1038/cddis.2014.470 25375377 PMC4260728

[26] Xu Y, Romero R, Miller D, Kadam L, Mial TN, Plazyo O, et al An M1-like macrophage polarization in decidual tissue during spontaneous preterm labor that is attenuated by rosiglitazone treatment. J Immunol. 2016;196(6):2476–2491. 10.4049/jimmunol.1502055 26889045 PMC4779725

[27] American College of Obstetrics and Gynecology Committee on Practice Bulletins-Obstetrics. ACOG Practice Bulletin Number 49, December 2003: Dystocia and augmentation of labor. Obstet Gynecol. 2003;102(6):1445–54. 14662243 10.1016/j.obstetgynecol.2003.10.011

[28] Blanc WA. Pathology of the placenta, membranes, and umbilical cord in bacterial, fungal, and viral infections in man. Monogr Pathol. 1981;(22):67–132. 7024790

[29] Ishida Y, Hayashi T, Goto T, Kimura A, Akimoto S, Mukaida N, et al Essential involvement of CX3CR1-mediated signals in the bactericidal host defense during septic peritonitis. J Immunol. 2008;181(6):4208–18. 18768878 10.4049/jimmunol.181.6.4208

[30] Wakabayashi A, Sawada K, Nakayama M, Toda A, Kimoto A, Mabuchi S, et al Targeting interleukin-6 receptor inhibits preterm delivery induced by inflammation. Mol Hum Reprod. 2013;19(11):718–26. 10.1093/molehr/gat057 23969038

[31] Robertson SA, Skinner RJ, Care AS. Essential role for IL-10 in resistance to lipopolysaccharide-induced preterm labor in mice. J Immunol. 2006;177(7):4888–96. 16982931 10.4049/jimmunol.177.7.4888

[32] Karjalainen MK, Ojaniemi M, Haapalainen AM, Mahlman M, Salminen A, Huusko JM, et al CXCR3 Polymorphism and Expression Associate with Spontaneous Preterm Birth. J Immunol. 2015;195(5):2187–98. 10.4049/jimmunol.1501174 26209629

[33] Nosaka M, Ishida Y, Kimura A, Hama M, Kawaguchi T, Yamamoto H, et al Immunohistochemical detection of intrathrombotic IL-6 and its application to thrombus age estimation. Int J Legal Med. 2015;129(5):1021–5. 10.1007/s00414-015-1147-9 25616628

[34] Nosaka M, Ishida Y, Kimura A, Kuninaka Y, Inui M, Mukaida N, et al Absence of IFN-γ accelerates thrombus resolution through enhanced MMP-9 and VEGF expression in mice. J Clin Invest. 2011;121(7):2911–20. 10.1172/JCI40782 21646723 PMC3223815

[35] Kobayashi A, Tanizaki Y, Kimura A, Ishida Y, Nosaka M, Toujima S, et al AG490, a Jak2 inhibitor, suppressed the progression of murine ovarian cancer. Eur J Pharmacol. 2015;766:63–75. 10.1016/j.ejphar.2015.09.039 26410360

[36] Gomez-Lopez N, StLouis D, Lehr MA, Sanchez-Rodriguez EN, Arenas-Hernandez M. Immune cells in term and preterm labor. Cell Mol Immunol. 2014;11(6):571–81. 10.1038/cmi.2014.46 24954221 PMC4220837

[37] Gonzalez JM, Franzke CW, Yang F, Romero R, Girardi G. Complement activation triggers metalloproteinases release inducing cervical remodeling and preterm birth in mice. Am J Pathol. 2011;179(2):838–49. 10.1016/j.ajpath.2011.04.024 21801872 PMC3157168

[38] Inui M, Ishida Y, Kimura A, Kuninaka Y, Mukaida N, Kondo T. Protective roles of CX3CR1-mediated signals in toxin A-induced enteritis through the induction of heme oxygenase-1 expression. J Immunol. 2011;186(1):423–31. 10.4049/jimmunol.1000043 21131421

[39] Siwetz M, Dieber-Rotheneder M, Cervar-Zivkovic M, Kummer D, Kremshofer J, Weiss G, et al Placental fractalkine is up-regulated in severe early-onset preeclampsia. Am J Pathol. 2015;185(5):1334–43. 10.1016/j.ajpath.2015.01.019 25769431 PMC4486762

[40] Kervancioglu Demirci E, Salamonsen LA, Gauster M. The role of CX3CL1 in fetal-maternal interaction during human gestation. Cell Adh Migr. 2016;10(1–2):189–96. 10.1080/19336918.2015.1089378 26745855 PMC4853041

[41] Shimoya K, Zhang Q, Tenma K, Ota Y, Hashimoto K, Shizusawa Y, et al Fractalkine (FRK) levels in amniotic fluid and its production during pregnancy. Mol Hum Reprod. 2003;9(2):97–101. 12569179 10.1093/molehr/gag009

[42] Nagamatsu T, Schust DJ. The immunomodulatory roles of macrophages at the maternal-fetal interface. Reprod Sci. 2010;17(3):209–18. 10.1177/1933719109349962 20065301

[43] Rinaldi SF, Catalano RD, Wade J, Rossi AG, Norman JE. Decidual neutrophil infiltration is not required for preterm birth in a mouse model of infection-induced preterm labor. J Immunol. 2014;192(5):2315–25. 10.4049/jimmunol.1302891 24501200 PMC3932811

[44] Gomaa MF, Serag Eldeen IF, Farid LA, El-Saeed MME, Abas AM, Aawd NM. Uterine natural killer cells dysregulation in idiopathic human preterm birth: a pilot study. J Matern Fetal Neonatal Med. 2017;30(15):1782–1786. 10.1080/14767058.2016.1224840 27593347

[45] Murphy SP, Hanna NN, Fast LD, Shaw SK, Berg G, Padbury JF, et al Evidence for participation of uterine natural killer cells in the mechanisms responsible for spontaneous preterm labor and delivery. Am J Obstet Gynecol. 2009;200(3):308.e1–9. 10.1016/j.ajog.2008.10.043 19114277 PMC3893044

[46] Rossi D, Zlotnik A. The biology of chemokines and their receptors. Annu Rev Immunol. 2000;18:217–42. 10.1146/annurev.immunol.18.1.217 10837058

[47] Ishida Y, Gao JL, Murphy PM. Chemokine receptor CX3CR1 mediates skin wound healing by promoting macrophage and fibroblast accumulation and function. J Immunol. 2008;180(1):569–79. 18097059 10.4049/jimmunol.180.1.569

[48] Furuichi K, Gao JL, Murphy PM. Chemokine receptor CX3CR1 regulates renal interstitial fibrosis after ischemia-reperfusion injury. Am J Pathol. 2006;169(2):372–87. 10.2353/ajpath.2006.060043 16877340 PMC1698788

[49] Combadière C, Potteaux S, Gao JL, Esposito B, Casanova S, Lee EJ, et al Decreased atherosclerotic lesion formation in CX3CR1/apolipoprotein E double knockout mice. Circulation. 2003;107(7):1009–16. 12600915 10.1161/01.cir.0000057548.68243.42

[50] Lesnik P, Haskell CA, Charo IF. Decreased atherosclerosis in CX3CR1^-/-^ mice reveals a role for fractalkine in atherogenesis. J Clin Invest. 2003;111(3):333–40. 10.1172/JCI15555 12569158 PMC151849

[51] Feng L, Chen S, Garcia GE, Xia Y, Siani MA, Botti P, et al Prevention of crescentic glomerulonephritis by immunoneutralization of the fractalkine receptor CX3CR1 rapid communication. Kidney Int. 1999;56(2):612–20. 10.1046/j.1523-1755.1999.00604.x 10432400

[52] Cook DN, Chen SC, Sullivan LM, Manfra DJ, Wiekowski MT, Prosser DM, et al Generation and analysis of mice lacking the chemokine fractalkine. Mol Cell Biol. 2001;21(9):3159–65. 10.1128/MCB.21.9.3159-3165.2001 11287620 PMC86945

[53] Jung S, Aliberti J, Graemmel P, Sunshine MJ, Kreutzberg GW, Sher A, et al Analysis of fractalkine receptor CX_3_CR1 function by targeted deletion and green fluorescent protein reporter gene insertion. Mol Cell Biol. 2000;20(11):4106–14. 10805752 10.1128/mcb.20.11.4106-4114.2000 PMC85780

[54] Sykes L, MacIntyre DA, Yap XJ, Teoh TG, Bennett PR. The Th1:th2 dichotomy of pregnancy and preterm labour. Mediators Inflamm. 2012;2012:967629 10.1155/2012/967629 22719180 PMC3376783

[55] Osman I, Young A, Ledingham MA, Thomson AJ, Jordan F, Greer IA, et al Leukocyte density and pro-inflammatory cytokine expression in human fetal membranes, decidua, cervix and myometrium before and during labour at term. Mol Hum Reprod. 2003;9(1):41–5. 12529419 10.1093/molehr/gag001

[56] Puchner K, Iavazzo C, Gourgiotis D, Boutsikou M, Baka S, Hassiakos D, et al Mid-trimester amniotic fluid interleukins (IL-1β, IL-10 and IL-18) as possible predictors of preterm delivery. In Vivo. 2011;25(1):141–8. 21282748

[57] Filipovich Y, Klein J, Zhou Y, Hirsch E. Maternal and fetal roles in bacterially induced preterm labor in the mouse. Am J Obstet Gynecol. 2016;214(3):386.e1–9. 10.1016/j.ajog.2015.10.014 26478101 PMC4775297

[58] Keelan JA, Marvin KW, Sato TA, Coleman M, McCowan LM, Mitchell MD. Cytokine abundance in placental tissues: evidence of inflammatory activation in gestational membranes with term and preterm parturition. Am J Obstet Gynecol. 1999; 181(6):1530–6. 10601939 10.1016/s0002-9378(99)70400-x

[59] Rueda CM, Presicce P, Jackson CM, Miller LA, Kallapur SG, Jobe AH, et al Lipopolysaccharide-induced chorioamnionitis promotes IL-1-dependent inflammatory Foxp3^+^ CD4^+^ T cells in the fetal rhesus macaque. J Immunol. 2016;196(9):3706–15. 10.4049/jimmunol.1502613 27036917 PMC4868637

[60] Sadowsky DW, Adams KM, Gravett MG, Witkin SS, Novy MJ. Preterm labor is induced by intraamniotic infusions of interleukin-1beta and tumor necrosis factor-alpha but not by interleukin-6 or interleukin-8 in a nonhuman primate model. Am J Obstet Gynecol. 2006;195(6):1578–89. 10.1016/j.ajog.2006.06.072 17132473

[61] Hirsch E, Filipovich Y, Mahendroo M. Signaling via the type I IL-1 and TNF receptors is necessary for bacterially induced preterm labor in a murine model. Am J Obstet Gynecol. 2006;194(5):1334–40. 10.1016/j.ajog.2005.11.004 16647919

[62] Toda A, Sawada K, Fujikawa T, Wakabayashi A, Nakamura K, Sawada I, et al Targeting Inhibitor of κB Kinase β Prevents Inflammation-Induced Preterm Delivery by Inhibiting IL-6 Production from Amniotic Cells. Am J Pathol. 2016;186(3):616–29. 10.1016/j.ajpath.2015.11.004 26796146

[63] Robertson SA, Skinner RJ, Care AS. Essential role for IL-10 in resistance to lipopolysaccharide-induced preterm labor in mice. J Immunol. 2006;177(7):4888–96. 16982931 10.4049/jimmunol.177.7.4888

[64] Li H, Zhou J, Wei X, Chen R, Geng J, Zheng R, et al miR-144 and targets, c-fos and cyclooxygenase-2 (COX2), modulate synthesis of PGE2 in the amnion during pregnancy and labor. Sci Rep. 2016;6:27914 10.1038/srep27914 27297132 PMC4906292

[65] Khanprakob T, Laopaiboon M, Lumbiganon P, Sangkomkamhang US. Cyclo-oxygenase (COX) inhibitors for preventing preterm labour. Cochrane Database Syst Rev. 2012;10:CD007748 10.1002/14651858.CD007748.pub2 23076936 PMC11403559

[66] Sykes L, MacIntyre DA, Teoh TG, Bennett PR. Anti-inflammatory prostaglandins for the prevention of preterm labour. Reproduction. 2014;148(2):R29–40. 10.1530/REP-13-0587 24890751

[67] Morita I. Distinct functions of COX-1 and COX-2. Prostaglandins Other Lipid Mediat. 2002;68–69:165–75. 12432916 10.1016/s0090-6980(02)00029-1

[68] Slater D.M., Dennes W.J., Campa J.S., Poston L. & Bennett P.R. Expression of cyclo-oxygenase types-1 and -2 in human myometrium throughout pregnancy. Mol Hum Reprod. 5, 880–4 (1999). 10460228 10.1093/molehr/5.9.880

[69] Slater DM, Dennes WJ, Campa JS, Poston L, Bennett PR. Expression of cyclo-oxygenase types-1 and -2 in human fetal membranes throughout pregnancy. Mol Hum Reprod. 1999;5(9):880–4. 10460228 10.1093/molehr/5.9.880

[70] Borna S, Saeidi FM. Celecoxib versus magnesium sulfate to arrest preterm labor: randomized trial. J Obstet Gynaecol Res. 2007;33(5):631–4. 10.1111/j.1447-0756.2007.00623.x 17845320

[71] Khanprakob T, Laopaiboon M, Lumbiganon P, Sangkomkamhang US. Cyclo-oxygenase (COX) inhibitors for preventing preterm labour. Cochrane Database Syst Rev. 2012;10:CD007748 10.1002/14651858.CD007748.pub2 23076936 PMC11403559

