# Supplementary figures and images for: Prevention of lipopolysaccharide-induced preterm labor by the lack of CX3CL1-CX3CR1 interaction in mice
Source: PLoS One. 2018 Nov 6;13(11):e0207085. doi: 10.1371/journal.pone.0207085 (PMC6219809; doi:10.1371/journal.pone.0207085)

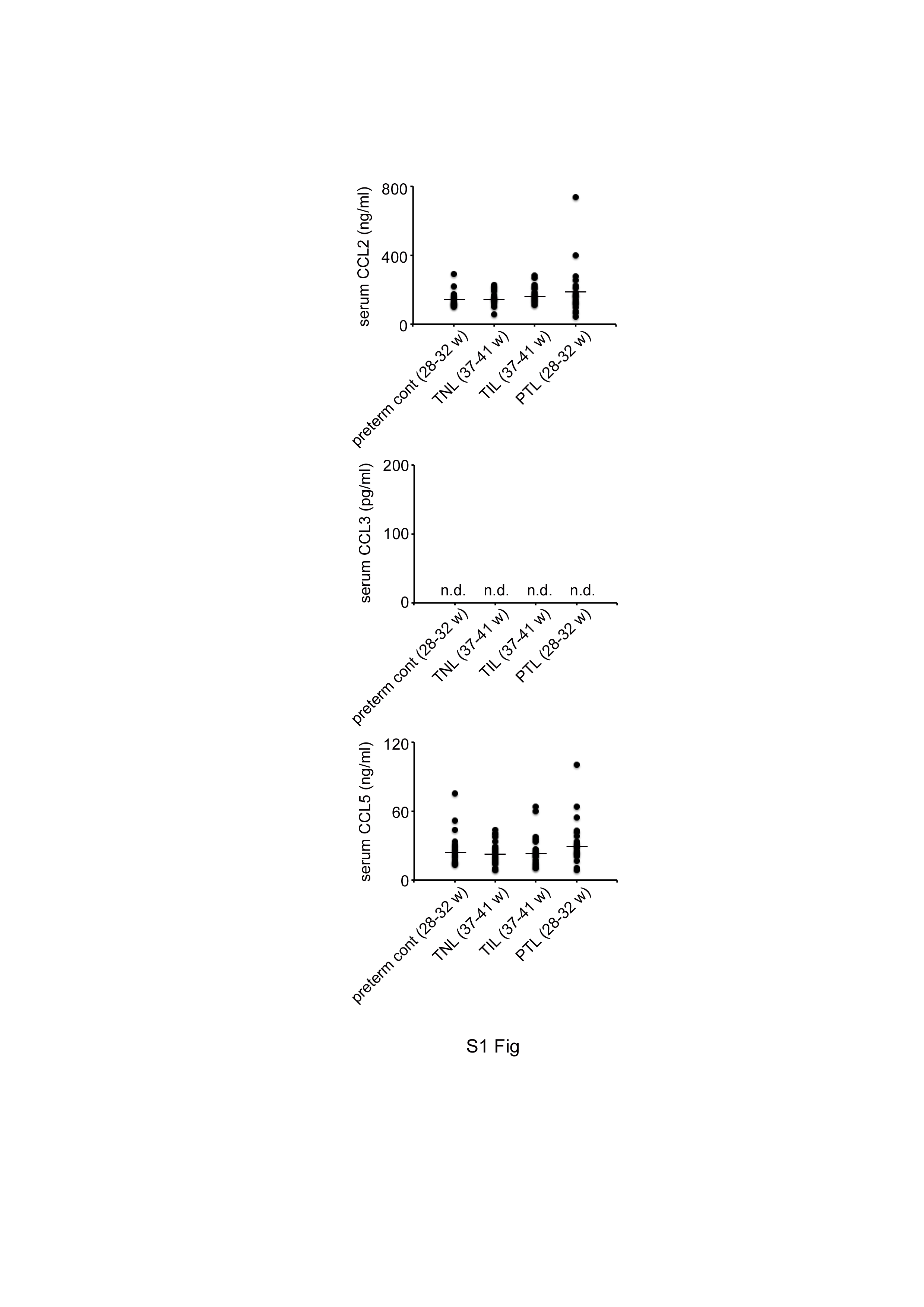

Supplement: S1 Fig — Human serum concentrations were determined on TNL, TIL, preterm control, and PTL groups (n = 20 in each group). (A) The serum CCL2 concentrations. (B) The serum CCL3 was not detected (n.d.) in all cases. (C) The serum CCL5 concentrations. Statistical significance determined using Mann-Whitney’s U test. *P < 0.05. (TIF) [file pone.0207085.s001.tif]

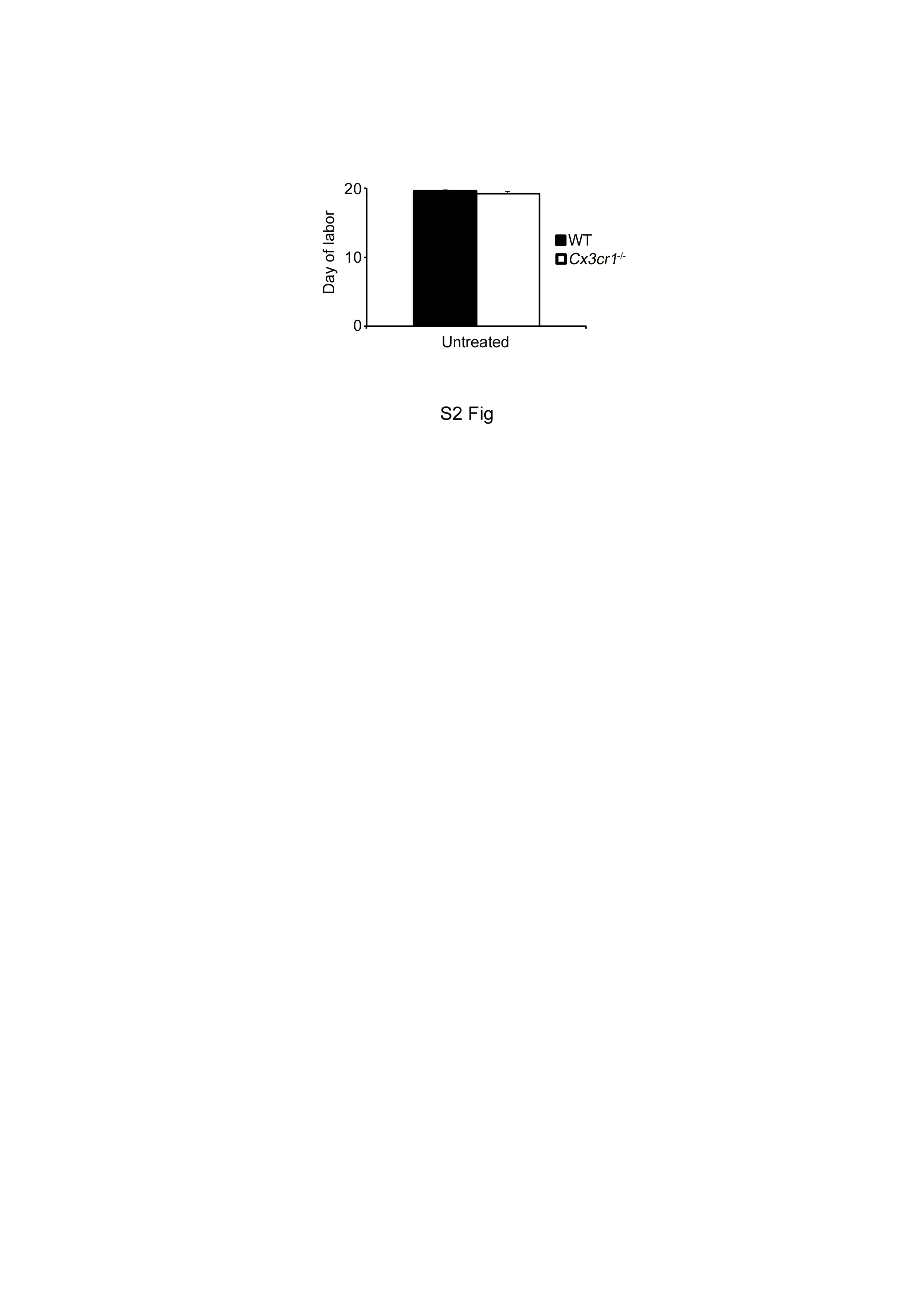

Supplement: S2 Fig — WT and Cx3cr1-/- mice labored at term when they were untreated (n = 5–8 in each group). (TIF) [file pone.0207085.s002.tif]

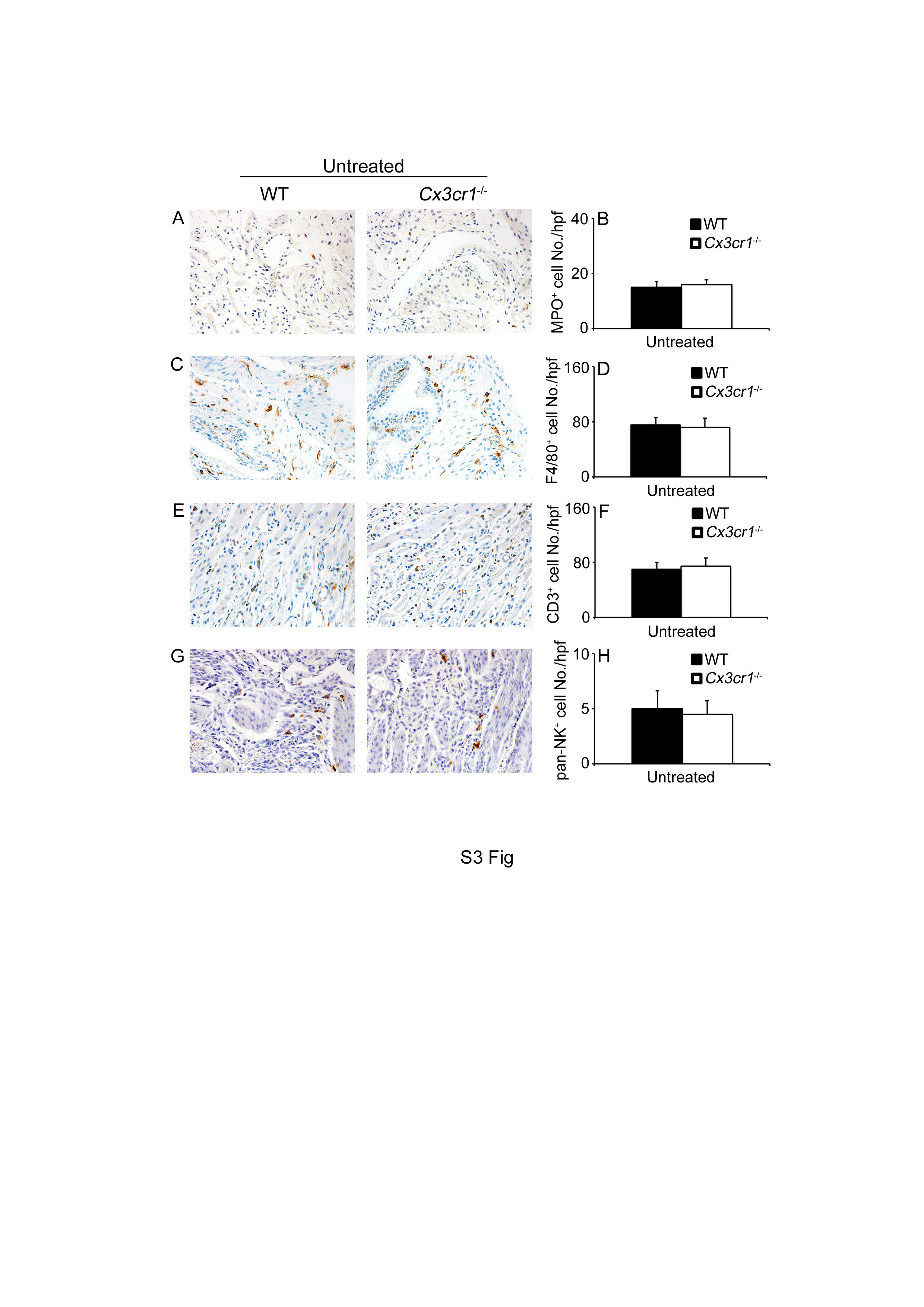

Supplement: S3 Fig — Evaluation of intrauterine leukocyte infiltration in WT and Cx3cr1-/- mice at gd 15.5 when they were untreated. Immunohistochemical analysis was performed using anti-MPO pAbs (A and B), anti-F4/80 mAb (C and D), anti-CD3 pAbs (E and F), and anti-pan NK cell mAb (G and H) as described in Materials and Methods. Representative results from 6 independent experiments are shown in panels A, C, E, and G. Original magnification, ×400. Enumeration of each leukocyte population including neutrophils (B), macrophages (D), T cells (F), and NK cells (H) in WT and Cx3cr1-/- mice with untreatment at gd 15.5. All values represent the mean ± SEM (n = 5–8 in each group). (TIF) [file pone.0207085.s003.tif]
